# Joint Acidosis and Acid-Sensing Receptors and Ion Channels in Osteoarthritis Pathobiology and Therapy

**DOI:** 10.3390/cells14201605

**Published:** 2025-10-16

**Authors:** William N. Martin, Colette Hyde, Adam Yung, Ryan Taffe, Bhakti Patel, Ajay Premkumar, Pallavi Bhattaram, Hicham Drissi, Nazir M. Khan

**Affiliations:** 1 Department of Orthopaedics, Emory Musculoskeletal Institute, Emory University, Atlanta, GA 30329, USA; billy.martin@emory.edu (W.N.M.); colette.hyde@emory.edu (C.H.); adam.yung@emory.edu (A.Y.); ryan.taffe@emory.edu (R.T.); bpatel331@gatech.edu (B.P.); ajay.premkumar@emory.edu (A.P.); pallavi.bhattaram@emory.edu (P.B.); hicham.drissi@emory.edu (H.D.); 2 College of Sciences, Georgia Institute of Technology, Atlanta, GA 30332, USA; 3 Joseph Maxwell Cleland Atlanta VA Medical Center, Decatur, GA 30033, USA

**Keywords:** osteoarthritis, acidosis, extracellular pH, GPR68/OGR1, GPR4, ASIC, TRPV1, cathepsin K, lactate, pH-responsive delivery

## Abstract

Osteoarthritis (OA) lacks disease-modifying therapies, in part because key features of the joint microenvironment remain underappreciated. One such feature is localized acidosis, characterized by sustained reductions in extracellular pH within the cartilage, meniscus, and the osteochondral interface despite near-neutral bulk synovial fluid. We synthesize current evidence on the origins, sensing, and consequences of joint acidosis in OA. Metabolic drivers include hypoxia-biased glycolysis in avascular cartilage, cytokine-driven reprogramming in the synovium, and limits in proton/lactate extrusion (e.g., monocarboxylate transporters (MCTs)), with additional contributions from fixed-charge matrix chemistry and osteoclast-mediated acidification at the osteochondral junction. Acidic niches shift proteolysis toward cathepsins, suppress anabolic control, and trigger chondrocyte stress responses (calcium overload, autophagy, senescence, apoptosis). In the nociceptive axis, protons engage ASIC3 and sensitize TRPV1, linking acidity to pain. Joint cells detect pH through two complementary sensor classes: proton-sensing GPCRs (GPR4, GPR65/TDAG8, GPR68/OGR1, GPR132/G2A), which couple to G_s_, G_q/11_, and G_12/13_ pathways converging on MAPK, NF-κB, CREB, and RhoA/ROCK; and proton-gated ion channels (ASIC1a/3, TRPV1), which convert acidity into electrical and Ca^2+^ signals. Therapeutic implications include inhibition of acid-enabled proteases (e.g., cathepsin K), pharmacologic modulation of pH-sensing receptors (with emerging interest in GPR68 and GPR4), ASIC/TRPV1-targeted analgesia, metabolic control of lactate generation, and pH-responsive intra-articular delivery systems. We outline research priorities for pH-aware clinical phenotyping and imaging, cell-type-resolved signaling maps, and targeted interventions in ‘acidotic OA’ endotypes. Framing acidosis as an actionable component of OA pathogenesis provides a coherent basis for mechanism-anchored, locality-specific disease modification.

## 1. Introduction

### Osteoarthritis and the Overlooked Axis of Joint Acidosis

Osteoarthritis (OA) is a heterogeneous whole-joint disease and a leading cause of disability worldwide [1,2,3]. Despite progress in phenotyping and risk-factor modification, there are still no approved disease-modifying osteoarthritis drugs (DMOADs), underscoring the need to define tractable pathogenic mechanisms [4,5]. This gap reflects disease heterogeneity and incomplete mapping of deep molecular mechanisms. Beyond structural degeneration, OA evolves within a metabolically constrained and inflamed microenvironment that alters fundamental physicochemical variables, including extracellular pH [6,7]. Accumulating evidence suggests that the OA joint is persistently acidic [8,9]. This phenomenon appears in the literature under several related terms—“joint acidosis,” “acidotic stress,” “extracellular acidification,” “acidic microenvironment”, and “low pH microenvironment”—and reflects a shift of extracellular pH (pHe) below the physiological range. Although tissue acidosis is well documented in chronic inflammatory arthritis such as rheumatoid arthritis (RA), with intra-articular pH values around ~6.6 recorded in vivo, it has received less systematic attention in OA, where reports vary in both magnitude and compartment measured [10,11,12]. Historical synovial fluid (SF) measurements showed that pH tracks local inflammatory activity and tends to be lower in RA than in OA.

In OA, early work on aspirated synovial fluid suggested only mild acidification relative to normal (means ~7.55 vs. ~7.77) [8], whereas recent chemical-exchange saturation transfer MRI with ultrashort echo time (acidoCEST-UTE) detects substantially lower pH within joint tissues [9]. For example, cartilage and meniscus pH have been estimated around 6.6–6.7, and in case-control imaging, the overall mean pH across the joint compartment is lower in OA than in non-OA knees (≈6.40 vs. 7.01) [9]. Taken together, the disparity between near-neutral synovial fluid values and clearly acidic cartilage/meniscus pH indicates that bulk fluid sampling underestimates the joint’s acid burden. This spatial blind spot helps explain prior inconsistencies and defines the key questions for OA: where and when acidosis arises, which cells sense it, and how it biases catabolism, inflammation, and pain. We therefore synthesize current evidence on acid generation, tissue effects, and proton-sensing pathways in joint cells, with implications for targeting this axis in OA. In this review, we focus on (i) the physiological basis of joint pH and a concise synthesis of reported pH ranges in OA and RA; (ii) biological sources of acidification in OA; (iii) how acidosis modulates chondrocyte and synovial cell programs (catabolism, survival, inflammation, nociception); and (iv) how joint cells sense acidic cues through acid-sensing G-protein-coupled receptors (GPR4, GPR65/TDAG8, GPR68/OGR1, GPR132/G2A) and ASIC channels, with an emphasis on signaling pathways that reprogram gene expression. We also consider proton-sensitive potassium K2P channels (TASK/TREK/TRAAK) as an acid–mechanics interface relevant to cartilage and synovium. We close by outlining therapeutic angles around pH buffering and receptor pharmacology relevant to OA.

## 2. Physiological Basis of Joint pH

### 2.1. What Has Been Measured in OA vs. RA

At baseline, knee synovial fluid (SF) is slightly more alkaline than blood and shows greater dispersion across individuals: In a classic series, venous blood averaged ~7.38, while normal SF averaged ~7.77 with a wide range (~6.5–8.9), indicating that “physiological” joint pH sits above systemic values and varies with local conditions [8]. In inflammatory disease, the apparent severity of acidosis depends strongly on both the compartment sampled and the method. In RA, direct in vivo microelectrode measurements inside the knee joint reported a mean pH around ~6.6, with values as low as ~6.0; in contrast, aspirated SF from the same joints often read closer to neutral, likely reflecting oxygenation and handling effects during ex vivo measurement [10,11]. In OA, older ex vivo SF studies suggested only a modest shift versus normal (e.g., ~7.55 in OA vs. ~7.77 in normal), and a later arthroplasty cohort reported a mean of 7.35 (range 6.80–7.68), again consistent with SF measurements under-reporting tissue acidity [8,13].

By contrast, tissue-level readouts consistently show an acidic milieu in OA joint structures. AcidoCEST-UTE MRI demonstrates cartilage at ~6.60 and meniscus at ~6.72, and in case–control imaging, the mean pH across regions of interest is lower in OA than in non-OA knees (≈6.40 ± 0.08 vs. 7.01 ± 0.26), with the OA meniscus being slightly higher than OA cartilage (*p* = 0.024) [9]. Earlier intraoperative microelectrode measurements summarized in Lombardi et al. also show a damage-related gradient in cartilage, from ~7.1 in grossly normal areas to ~5.5–6.2 in fissured/degenerate zones [9]. Collectively, these data support a spatially heterogeneous pH landscape in OA: cartilage and fibrocartilage are the most acidic, the osteochondral interface is likely acidic, and bulk SF can appear near neutral. Table 1 summarizes compartment-specific pH ranges across representative OA and RA studies.

From a signaling perspective, extracellular acidification is not merely a chemical context but an active cue that engages proton-sensing GPCRs (GPR4, GPR65/TDAG8, GPR68/OGR1, GPR132/G2A) and proton-gated ion channels (ASIC1a/3; TRPV1), which couple low pH to cAMP/PKA-CREB, Gq/PLC-MAPK, RhoA/ROCK, and NF-κB/AP-1 programs in joint cells [16,17,18,19,20].

### 2.2. Method/Compartment Considerations and Therapeutic Thresholds

Apparent discrepancies in “physiological” joint pH largely reflect what is sampled and how. Classic data show that normal SF averages are greater than blood pH (~7.7 vs. ~7.38), with wide dispersion [8], whereas direct in vivo microelectrodes in inflamed joints (RA) read acidic (~6.6; range to ~6.0) [11]. In OA, aspirated SF often appears near-neutral (e.g., mean ~7.35), but tissue-level imaging (acidoCEST-UTE) demonstrates acidic cartilage/meniscus and a lower mean pH in OA vs. non-OA knees [9,13]. Handling variables (temperature, CO_2_ outgassing, O_2_ equilibration, dwell time) bias SF upward toward neutrality, under-reporting tissue acidity [11]. For therapy design, this means that treatment thresholds should be defined by tissue-level pHe (or validated surrogates), not bulk SF alone; a baseline assumption of pHe ~7.0 would materially shift activation windows for pH-sensing targets, whereas the weight of evidence supports near-neutral SF but acidic tissue niches in OA [9,14].

## 3. Biological Sources of Acidosis in the Osteoarthritic Joint

### 3.1. Hypoxia-Driven Glycolysis in Articular Cartilage

**Figure 1:**
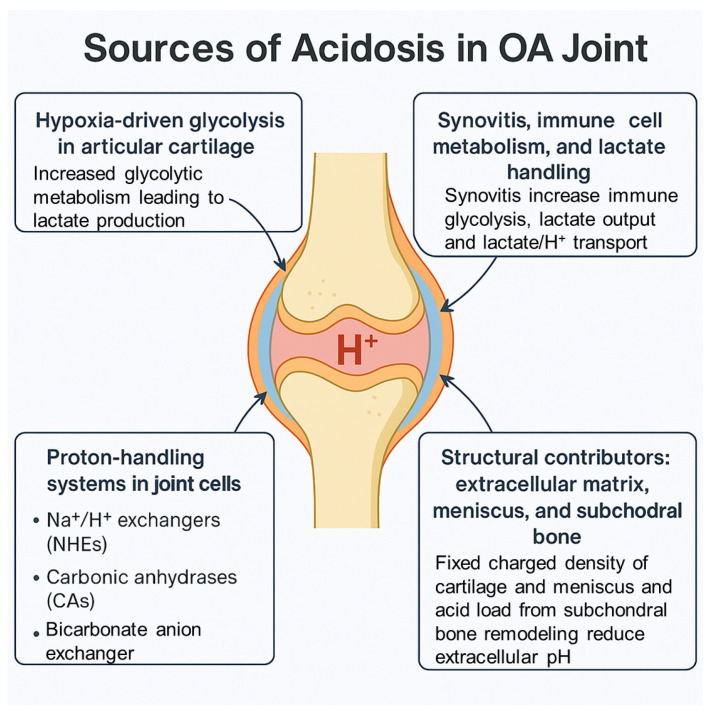
Sources of acidosis in the OA knee joint: A schematic illustration of an OA knee joint depicting major contributors to intra-articular acidosis. Key sources include (1) hypoxia-driven glycolysis in avascular cartilage leading to lactate accumulation; (2) immune-mediated glycolytic shift in the synovium and lactate/H^+^ efflux via monocarboxylate transporters (MCTs); (3) dysregulated proton-handling systems in joint cells, including Na^+^/H^+^ exchangers, carbonic anhydrases, and anion exchangers; and (4) structural contributors such as subchondral bone remodeling, meniscal degeneration, and loss of matrix fixed charge density that impair local buffering capacity.

Articular cartilage is avascular and operates at low oxygen tension; in OA, this hypoxic baseline is amplified by inflammatory signaling and altered mechanics, shifting chondrocyte metabolism toward glycolysis with increased lactate output [21,22,23]. Translational studies show that HIF-1α supports chondrocyte survival and promotes glycolytic gene programs, whereas HIF-2α (EPAS1) is induced by inflammatory cues and drives catabolic gene expression and apoptosis—changes linked to experimental OA progression [21,24,25,26,27]. Glycolytic reprogramming in OA chondrocytes is repeatedly observed: IL-1β/TNF exposure and abnormal loading increase glycolysis and lactate production; human OA chondrocytes under physiological compression demonstrate a metabolomic signature consistent with elevated glycolytic flux [7,23,28,29]. At the joint level, metabolomics of human OA synovial fluid (SF) frequently reports higher glycolytic intermediates and lactate (with heterogeneity across cohorts), consistent with a more hypoxic/acidotic milieu compared with non-OA joints (Figure 1) [30,31,32].

### 3.2. Synovitis, Immune-Cell Metabolism, and Lactate Handling

OA synovium is metabolically active and contributes to cytokine exposure of cartilage; synovitis is a recognized correlate of symptoms and structural progression [23]. Lactate efflux is a major determinant of extracellular proton load because monocarboxylate transporters (MCTs) co-transport lactate with H^+^ [33]. In RA synovium—where intra-articular acidosis is more pronounced—fibroblast-like synoviocytes up-regulate MCT4 (SLC16A3), and genetic or siRNA inhibition of MCT4 reduces inflammatory readouts and disease activity in vivo, establishing a mechanistic precedent for lactate-driven acidification of the joint space [34]. Direct stage-resolved evidence for MCT dysregulation in OA synovium remains limited and represents a gap for future work [23]. In chondrocytes, inflammatory stimulation increases LDHA-dependent lactate production and supports acid loading of the extracellular space, linking cytokine signaling to acidotic stress in OA cartilage [29,35] (Figure 1).

### 3.3. Proton-Handling Systems in Joint Cells

Chondrocytes and synoviocytes regulate intracellular/extracellular pH through Na^+^/H^+^ exchangers (NHEs), carbonic anhydrases (CAs), and bicarbonate/anion exchangers [36,37]. Human articular chondrocytes express NHE1–3 and adjust intracellular pH via NHE-dependent proton extrusion [36]. CA isoforms, including CA II/IX/XII, are present in cartilage and participate in CO_2_/HCO_3_^−^ buffering and pH homeostasis, particularly under hypoxia [38,39]. Hypoxia and inflammatory cues broadly up-regulate glucose transport and glycolytic genes in OA models, necessitating coordinated lactate/MCT-mediated export to limit intracellular acidosis [23,27] (Figure 1). A comprehensive, stage-specific atlas of these transporters and enzymes in OA joint tissues is still lacking [23].

### 3.4. Structural Contributors: Extracellular Matrix, Meniscus, and Subchondral Bone

The fixed negative charge density of the aggrecan-rich matrix shapes the local ionic milieu and facilitates slightly lower extracellular pH in cartilage; progressive proteoglycan loss in OA alters this physicochemical buffering capacity [40]. Meniscal fibrocartilage, which shares a high fixed-charge density, is likewise prone to acidic microdomains in OA, consistent with tissue-level pH mapping [40]. Subchondral bone remodeling adds another potential acid source: Osteoclast resorption requires V-ATPase-mediated proton secretion, and increased osteoclast activity is a feature of early OA [33,41] (Figure 1). Notably, osteoclasts can degrade cartilage through proteases without extracellular acidification in some contexts, indicating that acid-dependent and acid-independent pathways likely coexist at the osteochondral junction [42].

## 4. Consequences of Acidosis for Joint Tissues

### 4.1. Cartilage: Matrix Catabolism, Loss of Anabolism, and Stress Responses

**Figure 2:**
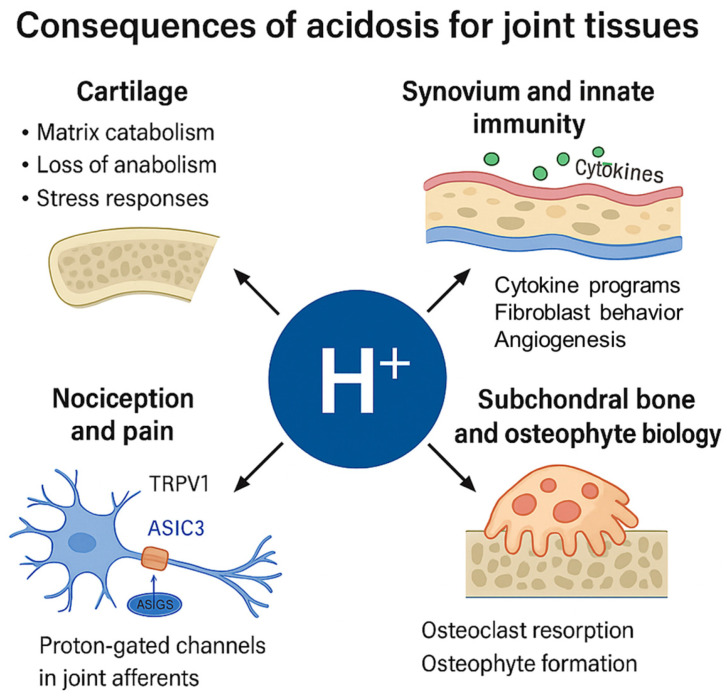
Consequences of joint acidosis on tissue compartments: Visual summary of tissue-specific effects of extracellular acidosis in OA. In cartilage, acidic pH favors cathepsin activity, reduces SOX9 expression, and induces ASIC1a-mediated apoptosis. In the synovium, acidosis supports fibroblast migration, cytokine release, and angiogenesis. In afferent nerves, protons activate ASIC3 and TRPV1 to mediate nociception and pain. In subchondral bone, osteoclast-driven acid secretion enhances resorption and may promote osteophyte formation.

A fall in extracellular pH (pHe) modifies the balance of cartilage proteolysis by favoring cysteine cathepsins, whose activities peak at acidic pH, while most MMPs work best near neutral pH [43,44]. In OA cartilage and synovium, cathepsin K is induced and can cleave type II collagen and aggrecan under acidic conditions, implicating acid-enabled cathepsin activity in matrix loss [15,45,46]. Acid exposure also perturbs chondrocyte gene programs: In controlled culture, lowering pHe suppresses SOX9 and VEGF transcripts while leaving COL2A1/ACAN relatively unchanged, indicating direct pH sensitivity of the chondrocyte transcriptional network [47] (Figure 2).

Protons act as signals via ASIC1a in chondrocytes, where acidification triggers Ca^2+^ influx, mitochondrial stress, and apoptosis; pharmacologic or genetic ASIC1a inhibition reduces acid-induced death [16,48]. Pro-inflammatory cytokines further augment acid toxicity by up-regulating ASIC1a, linking inflammation to heightened proton sensitivity [49]. Beyond apoptosis, acid–ASIC1a signaling promotes autophagy and features of senescence [16,50]. Collectively, these data support a model in which localized acidosis tilts protease usage toward cathepsins, attenuates anabolic control (SOX9), and activates ASIC1a-dependent stress pathways in chondrocytes [43,47,48].

### 4.2. Synovium and Innate Immunity: Cytokine Programs, Fibroblast Behavior, and Angiogenesis

OA synovial fluid provides a milieu that favors M1-skewed macrophage programs associated with inflammatory mediators, linking synovitis to symptoms and structural changes [51,52,53] (Figure 2). Direct pH-specific evidence in OA synovium is limited; however, in RA synovium, extracellular acidosis (≈pH 6.8) enhances fibroblast-like synoviocyte (FLS) migration and Na^+^/HCO_3_^−^ transporter activity, supporting acid-driven tissue invasiveness [54]. Acidic conditions also stimulate FLS release of VEGF and promote microvascular formation in arthritic tissue models, pointing to an angiogenic effect of acidosis in inflamed synovium [55]. Extrapolating to OA, these findings suggest that acidic niches in OA synovium could reinforce macrophage-mediated inflammation and fibroblast activation, although direct OA pH manipulation studies are still needed [51,54].

### 4.3. Nociception and Pain: Proton-Gated Channels in Joint Afferents

Protons activate joint nociceptors via ASIC3, producing rapid neuronal depolarization and action potential firing; sustained exposure and channel cross-talk (e.g., TRPV1) can further amplify pain signaling [17]. Human and mouse data indicate that lysophosphatidylcholine 16:0 (LPC16:0), enriched in painful joints, drives chronic pain through ASIC3-dependent peripheral input and spinal sensitization, with relevance to OA cohorts [56]. TRPV1 is expressed in joint tissues and nociceptors; clinical and preclinical work implicates TRPV1 in OA pain, and mechanistic studies suggest TRPV1 activity can also modulate synovial inflammation [18,57,58]. Together, these observations place proton-gated ASIC3 and acid-sensitive TRPV1 as key conduits from acidic microenvironments to pain signaling in OA [17,56] (Figure 2).

### 4.4. Subchondral Bone and Osteophyte Biology

Osteoclast resorption requires vacuolar V-ATPase–mediated proton secretion, generating highly acidic lacunae that enable cathepsin K activity [43,44]. In OA, subchondral bone remodeling and angiogenesis emerge early and contribute to cartilage degeneration and pain [41]. Osteoclast-initiated remodeling promotes sensory innervation of subchondral bone via netrin-1, providing a mechanistic link between bone turnover and pain [59]. Pharmacologic cathepsin K inhibition (MIV-711) reduces subchondral bone turnover and attenuates cartilage pathology in animal OA, and in phase 2a human studies slows structural progression despite limited symptomatic change, underscoring the pathogenic contribution of acid-enabled osteoclastic proteolysis [60,61] (Figure 2).

### 4.5. What Is Established in RA vs. Still Emerging in OA

Classic clinical studies show markedly lower synovial pH in RA than in OA, with RA SF often near pH 6.6–7.0, whereas OA SF is closer to blood pH with wider dispersion [10,11]. RA FLS display acid-enhanced migration and angiogenic signaling, directly tying acidosis to synovial pathology [54,55]. In OA, in vivo pH mapping is sparse; nevertheless, OA tissues exhibit acid-enabled cathepsin activity, chondrocyte ASIC1a-dependent stress responses, ASIC3-linked pain behaviors, and bone remodeling-associated nociception, together supporting biologically meaningful acidic niches despite less dramatic bulk SF acidosis than RA [15,17,41,48,59].

## 5. How Joint Cells Sense Acidity

### 5.1. Proton-Sensing G-Protein-Coupled Receptors (GPCRs)

**Figure 3:**
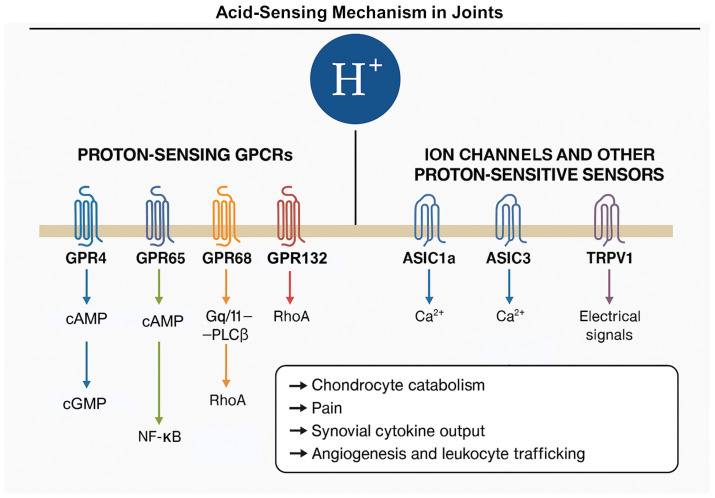
Acid-sensing mechanisms in joint cells: Joint cells deploy two primary classes of acid sensors. Left panel: Proton-sensing G-protein-coupled receptors (GPCRs), including GPR4, GPR65 (TDAG8), GPR68 (OGR1), and GPR132, couple to Gs, Gq/11, or G12/13 pathways, modulating cAMP, PLCβ, RhoA, and NF-κB signaling. Right panel: Acid-sensing ion channels (ASIC1a, ASIC3) and TRPV1 transduce protons into calcium influx or electrical signals, mediating stress responses and nociception. These pathways regulate catabolism, inflammation, angiogenesis, and pain in OA.

A small class of class-A GPCRs—GPR4, GPR65 (TDAG8), GPR68 (OGR1), and GPR132 (G2A)—detect extracellular protons through titratable histidines in their extracellular domains and gate downstream signaling over a physiological pH window (≈5.5–7.8) [19,20] (Figure 3). These receptors show distinct coupling preferences: GPR4 predominantly engages Gs-cAMP with downstream EPAC/PKA and NF-κB; GPR68 couples to Gq/11-PLCβ-Ca^2+^/PKC and can also signal via RhoA; GPR65 largely raises cAMP and dampens NF-κB-driven inflammation in immune cells; GPR132 has more context-dependent coupling and is also responsive to oxidized lipids [20,62,63,64,65,66].

#### 5.1.1. Evidence in OA Tissues

GPR68 (OGR1) is robustly expressed in human OA cartilage, correlates with matrix degeneration, and its pharmacologic activation in human chondrocytes suppresses MMP expression under IL-1β stimulation, pointing to a catabolism-restraining role [67]. Mechanistically, GPR68 integrates proton sensing with membrane stretch, a property relevant to mechanically loaded cartilage [68].

GPR4 is up-regulated in human and murine OA cartilage; genetic or experimental modulation of GPR4 alters OA outcomes (cartilage degeneration, synovitis, osteophytes, and pain) via a CXCL12/CXCR7–NF-κB/MEK axis [69]. In endothelial cells, acidosis → GPR4 signaling increases leukocyte adhesion and induces inflammatory genes through cAMP/EPAC and NF-κB, supporting a role in leukocyte trafficking to acidic synovium [62,63]. GPR4 couples acidosis to cAMP/EPAC → NF-κB and adhesion gene induction (e.g., ICAM1/VCAM1/SELE) in vascular/immune contexts [69,70]. These data suggest the context-dependent roles of GPR4 in joint pathology.

For GPR65 (TDAG8), multiple immune-inflammation models indicate anti-inflammatory actions of proton-activated signaling (increased cAMP; reduced cytokines), although direct OA-synovium datasets remain limited [20,64,66]. GPR65 generally attenuates inflammation via cAMP in immune cells, but in the synovial fibroblast niche can contribute to inflammatory joint pain, highlighting cell-type-specific polarity under modest pH decrements. These data reinforce the need for compartment-aware targeting and local delivery [71].

For GPR132 (G2A), early reports suggested proton sensitivity, but accumulating evidence emphasizes lipid ligands (e.g., LPC) and cross-talk with nociceptive channels; G2A activation can sensitize TRPV1 via PKC in sensory neurons, linking acidic/lipidic milieus to pain [20,65,72].

Across these receptors, proton sensing feeds into MAPK, NF-κB, CREB, and RhoA/ROCK programs that regulate chondrocyte catabolism, synovial cytokine output, angiogenesis, and leukocyte trafficking–signaling axes directly implicated in OA pathophysiology [20,62,63,64] (Figure 3).

#### 5.1.2. Expression Landscape Across OA-Relevant Cell Types

Chondrocytes. Bulk and single-cell data show expression of GPR68 and GPR4, with ASIC1a/ASIC3 and TRPV1 variably detected; in OA cartilage, GPR68 expression correlates with matrix degeneration, and GPR68 activation can suppress catabolic gene expression (e.g., MMP-13) in inflamed chondrocytes [67]. GPR4 is detectable in human chondrocytes and rises with stressors (e.g., AGEs), with functional studies implicating CXCL12/CXCR7 signaling; pharmacologic antagonism (NE 52-QQ57) mitigates OA features in preclinical and ex vivo settings [70].

Synovial fibroblasts (SFs). Single-cell and bulk transcriptomics show distinct SF subsets associated with inflammation, matrix remodeling, and pain in arthritis; in knee OA, tissue from patient-reported pain sites exhibits a differential SF phenotype with pathway enrichment in fibrosis/inflammation/neuronal growth [73]. The broader RA framework demonstrates anatomically and functionally distinct SF populations driving inflammation vs damage [74], a template likely relevant to pH-sensor heterogeneity in OA synovium. Importantly, new mechanistic work shows acid-sensing GPCR expression and function in human/mouse FLS: GPR65 activation on FLS contributes to inflammatory joint pain, linking low pHe to FLS–neuron signaling [71]. Together, these data support evaluating GPR4/65/68/132 (and K2P) across SF subsets as potential pH-responsive drivers of synovitis and pain [71,73,74].

Immune cells. Proton-sensing GPCRs are expressed in inflammatory myeloid and lymphoid compartments, with GPR65 often dampening NF-κB-dependent cytokines via cAMP signaling, whereas GPR4/GPR68 can promote adhesion and inflammatory programs in endothelium/immune contexts. In OA, stage-resolved immune-cell maps with pHe metadata remain limited, but the combination of acidic SF niches and infiltrating immune cells provides a plausible route for cell-type-specific pH-sensor actions [20].

Sensory neurons. Beyond ASIC3/TRPV1, acid-sensing GPCRs are present in nociceptors: in dorsal root ganglia, proton-sensing GPCR expression is dynamically regulated by inflammatory stimuli, with increased TDAG8 (GPR65) expression after peripheral inflammation [75]. These GPCRs, together with ASIC/TRPV1, provide parallel conduits by which acidic microdomains in the joint can drive peripheral nociception [75].

Sensor redundancy and context. Because multiple acid sensors coexist within a single joint and sometimes within the same cell, outputs can be redundant or opposing depending on pHe, load, ligand bias, and cell state. Therefore, co-expression of GPCRs (GPR4/65/68/132), ion channels (ASIC1a/3; TRPV1), and K2P channels (TASK/TREK/TRAAK) within a joint creates redundant, sometimes opposing outputs governed by local pHe, mechanical load, ligand bias, and cell state. For example, GPR65 tends to temper cytokine production in immune cells, while GPR4 can amplify adhesion/inflammatory genes in endothelium and is upregulated in OA cartilage (see 6.2), whereas GPR68 shows anti-catabolic effects in OA chondrocytes but can be pro-inflammatory in other cell types. This cell-type-specific polarity underlines the need for pH-aware, compartment-targeted strategies [20,75].

### 5.2. Ion Channels and Other Proton-Sensitive Sensors

Acid-sensing ion channels (ASICs): ASIC1a on articular chondrocytes mediates Ca^2+^ influx, mitochondrial stress, autophagy, senescence, and apoptosis under acidic conditions; pharmacologic blockade (PcTx-1) or genetic inhibition mitigates these responses [16,50,53,76].

ASIC3 on joint afferents links protons to OA pain; in the rat MIA-OA model, the selective ASIC3 peptide blocker APETx2 reduces pain and—when given early—limits cartilage damage, connecting nociceptive input to disease modification [17] (Figure 3).

TRPV channels. TRPV1 is present in joint tissues and nociceptors; acidic milieus sensitize TRPV1, and increased TRPV1 activity is documented in human OA synovium and in rat OA models [18]. While systemic TRPV1 antagonists have faced hyperthermia liabilities, local or peripherally restricted approaches remain under investigation [77].

In summary, joint cells deploy two complementary sensor classes for acidosis: Proton-sensing GPCRs that transduce pH shifts into gene-regulatory programs (cAMP/EPAC, PLC–MAPK, NF-κB, RhoA/ROCK), and ASIC/TRPV channels that transduce protons into electrical/Ca^2+^ signals driving stress responses and pain. In OA, the most direct human evidence currently points to GPR4 and GPR68 in cartilage, ASIC1a in chondrocytes, and ASIC3/TRPV1 in nociception; GPR65 likely modulates immune tone, with G2A contributing through lipid–acid cross-talk. Priorities include cell-type-resolved maps across OA stages and perturbation studies in human tissue to define receptor- and channel-specific therapeutic windows [16,17,67,69].

### 5.3. Downstream Signaling Programs and OA Readouts

**Figure 4:**
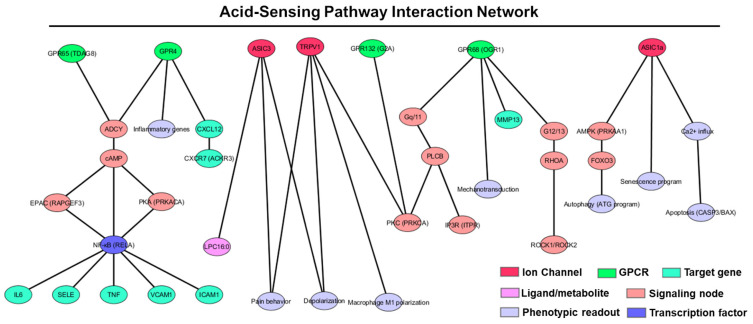
Acid-sensing pathway interaction network in OA: Network representation of downstream signaling cascades triggered by proton-sensing GPCRs (GPR4, GPR65, GPR68, GPR132) and ion channels (ASIC1a, ASIC3, TRPV1) in joint cells. These sensors engage distinct signaling nodes (cAMP, PLCβ, RhoA, Ca^2+^) that converge on transcription factors (NF-κB, FOXO3) and regulate OA-relevant genes (e.g., IL-6, TNF, MMP13, CXCL12). Phenotypic outputs include inflammation, catabolism, pain, autophagy, and senescence. Node types are color-coded by function: ion channels (red), GPCRs (green), ligands/metabolites (pink), transcription factors (blue), phenotypic outputs (lavender), signaling intermediates (light red), and target genes (cyan).

Proton-sensing GPCRs couple to Gs/cAMP–PKA–CREB (e.g., GPR65), Gq/PLC–MAPK (e.g., GPR68), and G12/13–RhoA/ROCK (reported across the family), while ASIC1a/3 and TRPV1 drive Ca^2+^ influx and membrane depolarization (Figure 4). Across joint cell types, these inputs converge on MAPK and NF-κB/AP-1 to regulate MMP-13, ADAMTS, IL-6/IL-8, COX-2/PGE_2_, and survival programs.

GPR4 activates ADCY → cAMP → EPAC/PKA, driving NF-κB and endothelial adhesion genes (ICAM1, VCAM1, SELE) and can upregulate CXCL12 in OA models [62,63,69]. GPR68 engages Gq/11 → PLCB → IP3/Ca^2+^ → PKC → ERK and G12/13 → RHOA/ROCK, integrates membrane stretch, and suppresses MMP13 in human OA chondrocytes when potentiated by the PAM ogerin [67,68,78]. GPR65 elevates cAMP and typically dampens NF-κB-dependent cytokines (e.g., IL-6, TNF) in immune cells [64,66]. GPR132 can signal through PKC and sensitize TRPV1, linking acidic/lipidic milieus to nociception [65,72]. On the ion-channel side, ASIC1a triggers Ca^2+^ influx, AMPK–FOXO3-dependent autophagy, senescence features, and apoptosis programs in chondrocytes, whereas ASIC3 and TRPV1 transduce acidity into peripheral nociception; TRPV1 also modulates macrophage polarization in synovium [16,17,18,50,58,76] (Figure 4). These convergent pathways (MAPK, NF-κB, CREB, RhoA/ROCK) provide gene-level handles for intervention and for biomarker development.

Although GPR4/65/68/132 and ASICs can show basal activity near physiological pH, several features explain how modest pH decrements (e.g., 7.4 → 7.0 or 7.2 → 6.8) can amplify OA-relevant signaling. First, their pH–activity relations are steep within the physiologic-to-acidic window (e.g., GPR4/TDAG8/GPR68 show pronounced activation from ~7.8–6.5; ASICs gate over ~7.2–6.0), so small ΔpH can yield large Δsignal [19,20,66]. Second, acidic microdomains form within cartilage/meniscus and at the osteochondral interface, so local pHe can be well below bulk SF [9]. Third, receptor/channel reserve and biased coupling (e.g., GPR68 Gq vs. cAMP arms; ASIC subtype composition) allow partial activation to drive MAPK, NF-κB, CREB, or RhoA/ROCK outputs that control MMP-13, IL-6, PGE_2_, and survival programs. Finally, mechanochemical coincidence—demonstrated for GPR68—means pH and stretch together boost gain, aligning with load-bearing acidic niches in OA [68,79].

### 5.4. Proton-Sensitive K2P Potassium Channels (TASK/TREK/TRAAK) in the Acid–Mechanics Interface

Two-pore domain K^+^ (K2P) channels integrate protons and mechanical stimuli, positioning them alongside GPR68 as coincidence detectors relevant to joint microenvironments. TASK-1 (KCNK3) and TASK-3 (KCNK9) are inhibited by extracellular acidosis (pKa near the physiological range), whereas alkalinization relieves block; recent structural/biophysical work refines these pH set points (∼7.4 for TASK-1; ∼6.7 for TASK-3) [80,81]. TASK-2 (KCNK5) couples extracellular pH to chondrocyte membrane potential and has been proposed to link pHe to chondrocyte activity [82]. TREK-1/2 (KCNK2/KCNK10) and TRAAK (KCNK4) are mechanosensitive channels modulated by pH and membrane lipids; extracellular acidification can strongly inhibit TREK-1 with an apparent pK ≈ 7.4, while intracellular acidosis and physical stimuli (stretch, osmotic changes) can activate TREK-family currents [83,84,85]. In cartilage, chondrocytes express K2P channels, and recent work demonstrated TREK-family expression and function in rat articular chondrocytes, implicating these channels in osmotic mechanotransduction and ECM integrity [86,87,88]. Conceptually, acidosis that inhibits TASK or alters TREK gating will depolarize chondrocytes, reshape Ca^2+^ entry, and modulate downstream transcription (e.g., MAPK/NF-κB), thereby complementing proton-sensing GPCR and ASIC pathways in OA.

## 6. Therapeutic Implications of Joint Acidosis

### 6.1. Protease Axis: Cathepsin K and Acid-Enabled Matrix Degradation

**Figure 5:**
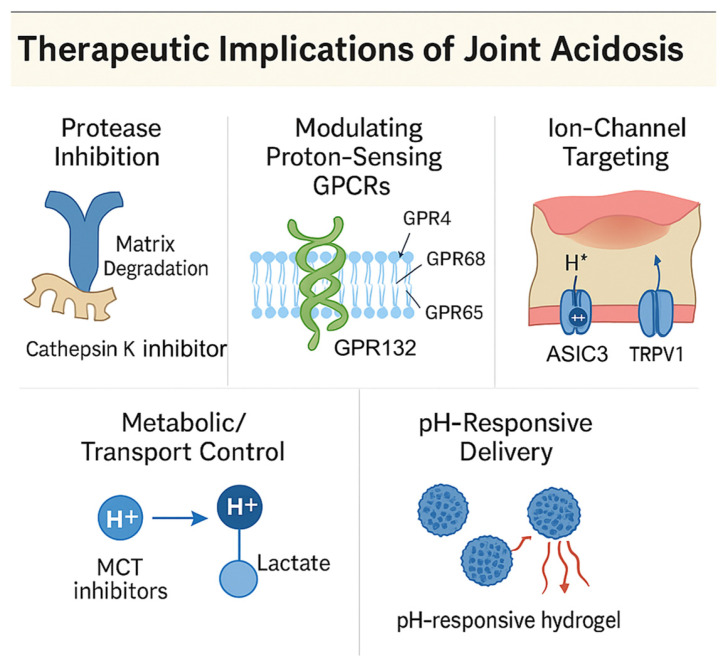
Therapeutic strategies targeting joint acidosis: Overview of emerging therapeutic modalities directed at the acidosis axis in OA. Approaches include (1) protease inhibition via cathepsin K antagonists; (2) pharmacological modulation of proton-sensing GPCRs (e.g., GPR4 antagonists, GPR68 allosteric modulators); (3) blockade of ion channels (ASIC1a, ASIC3, TRPV1); (4) targeting acid generation and transport via LDHA and MCT inhibitors; and (5) use of pH-responsive hydrogels or nanoparticles for localized delivery. These strategies aim to modulate inflammation, cartilage degradation, and pain.

Cathepsin K requires an acidic milieu for optimal collagenolysis; inhibiting this protease has been explored as a disease-modifying strategy in OA [43]. In a randomized phase 2a study, the cathepsin-K inhibitor MIV-711 produced structural benefits—less medial femoral bone-area expansion and reduced cartilage thinning on quantitative MRI—without significant pain improvement over 26 weeks [60,89]. These data support acid-enabled cathepsin activity as a relevant target but also highlight the common dissociation between structure and symptoms in OA trials [60,89] (Figure 5).

### 6.2. Modulating Proton-Sensing GPCRs

GPR4. Acidosis–GPR4 signaling induces endothelial adhesion molecules and broad inflammatory gene programs via cAMP/EPAC → NF-κB; small-molecule GPR4 antagonists suppress these responses in vitro, nominating GPR4 as a druggable node of acid-driven inflammation [62,63]. In OA models, GPR4 is up-regulated in cartilage, and genetic/experimental modulation alters cartilage degeneration, synovitis, osteophytes, and pain through a CXCL12/CXCR7–NF-κB/MEK axis, suggesting translational relevance to joint pathology [69]. Pharmacologic GPR4 antagonism (NE 52-QQ57) suppresses AGE-induced cartilage degradation and inflammatory outputs in human explants/lines, nominating GPR4 as a joint-relevant, drug-gable node [69,70].

GPR68 (OGR1). Medicinal chemistry work shows ogerin and related PAMs potentiate GPR68 responses in a pH window (~6.5–7.4) that overlaps measured OA tissue pH, suggesting that intra-articular delivery of a GPR68 PAM might bias stressed chondrocytes toward lower catabolic output while preserving proton-dependent gating [78]. In primary human OA chondrocytes, pharmacologic activation of GPR68 with the positive allosteric modulator ogerin reduces MMP-13 expression under IL-1β stimulation, indicating suppression of a key catabolic effector [67]. Because MMP-13 is the principal collagenase for type II collagen in OA cartilage, these in vitro data support the hypothesis that enhancing GPR68 signaling could be chondroprotective in vivo, warranting dedicated translational studies [67]. Notably, GPR68 behaves as a mechanochemical sensor, integrating membrane stretch with proton sensing—relevant for mechanically loaded cartilage [68]. Given GPR68’s ability to couple G_q/11_ and G_12/13_ and to activate MAPK/RhoA programs, cell-type-specific effects in synovium and vasculature remain possible; therefore, local delivery and careful pharmacodynamic monitoring are advisable as this axis moves toward therapy [20].

GPR65 (TDAG8). Proton-activated GPR65 generally raises cAMP and dampens NF-κB in immune cells; multiple inflammatory models support anti-inflammatory actions [64,66]. However, recent human/mouse data show GPR65 on synovial fibroblasts contributes to inflammatory joint pain (and neuron-FLS crosstalk), implying that cell-type context determines net phenotype. While OA-targeted small molecules are not yet available, the FLS-pain biology motivates exploration of local modulation or biased signaling approaches [71]. Emerging human genetics and chemical biology show that modulating GPR65 can reshape cytokine networks, making it a plausible lever for synovial inflammation in acidic niches, though OA-specific pharmacology is still lacking [90].

### 6.3. Ion Channels: ASIC and TRPV1 Approaches

ASIC3 antagonism reduces OA pain and can attenuate structural damage when administered early: In the rat MIA model, intra-articular APETx2 diminished weight-bearing pain and limited cartilage loss, linking proton-driven nociception to disease modification [17]. ASIC1a blockade (e.g., PcTx-1; small-molecule tools) protects chondrocytes from acid-induced Ca^2+^ overload, mitochondrial stress and apoptosis in vitro and ex vivo, supporting ASIC1a as a cartilage-protective target [76].

TRPV1 remains attractive for analgesia but systemic antagonists (e.g., AMG-517, AZD1386) produced hyperthermia in clinical trials, motivating peripherally restricted or local strategies [91,92,93]. One alternative is resiniferatoxin (RTX), a TRPV1 agonist that ablates nociceptors; early intra-articular RTX trials in knee OA show feasibility and analgesic signals, and phase 3 studies (NCT04044742; NCT04885972) are underway [94].

### 6.4. Metabolic and Transport Interventions to Reduce Acid Load

In chondrocytes, LDHA promotes ROS-dependent catabolic signaling; LDHA inhibition (e.g., FX11) reduces OA pathology in preclinical models, positioning glycolytic control upstream of acid generation [35]. Targeting lactate transport is another lever: MCT1/4 inhibitors are in early-phase oncology trials and conceptually could limit proton-coupled lactate export from glycolytic joint cells, though no OA trials exist and safety/tissue-specificity are open questions [95,96]. Given the widespread roles of MCTs, local delivery would likely be required [95] (Figure 5).

### 6.5. pH-Aware Delivery and Patient Selection

Acidic niches in OA can be exploited for delivery using pH-responsive hydrogels and nanoparticles that release cargo preferentially at low pH; several platforms improve intra-articular residence and demonstrate cartilage protection in OA models, although no pH-responsive system is yet approved (Figure 5) [97,98,99]. Because bulk synovial fluid pH can appear near neutral while tissues are acidic, patient stratification and pharmacodynamic imaging will be important. Noninvasive pH-mapping methods (e.g., acidoCEST-UTE MRI) offer a route to enrich for “acidotic OA” endotypes and to monitor target engagement in trials [9].

Collectively, the pipeline spans protease inhibition, proton-sensor modulation, ASIC/TRPV1 nociceptor targeting, metabolic control of lactate/acid generation, and pH-responsive delivery. The most advanced DMOAD-like signal to date is cathepsin-K inhibition with structural benefit absent symptom change [60]. For proton sensors and ASIC/TRPV1, proof-of-concept is strong preclinically, and clinical positioning now hinges on safety (TRPV1) and selective, joint-localized delivery (ASIC/GPCR modulators) (Figure 5) [17,92,94].

Table 2 summarizes therapeutic classes that engage the acid axis in OA—protease inhibition, proton-sensor modulation, ion-channel targeting, metabolic/transport control, and pH-responsive delivery—along with stage of evidence and key risks [17,35,60,62,63,67,69,76,78,91,92,95,96,97,98,99].

## 7. Knowledge Gaps and Research Priorities

### 7.1. Quantifying Joint Acidosis In Vivo (Where, When, and How Much?)

**Figure 6:**
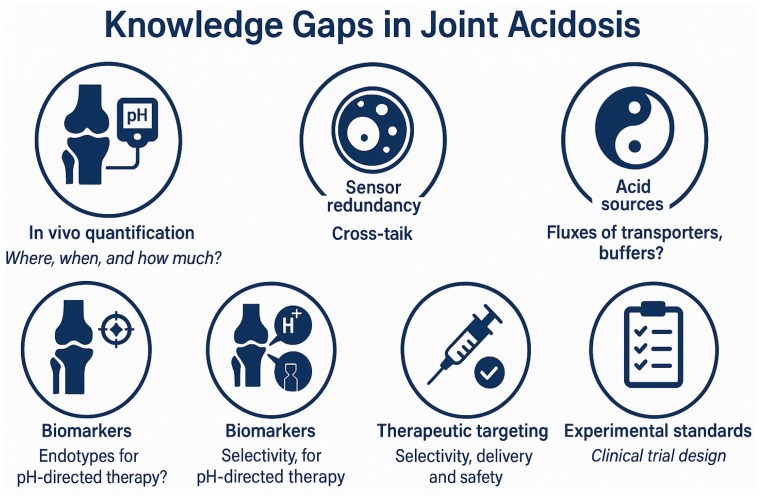
Knowledge gaps in the study of joint acidosis: Summary of seven key areas requiring further investigation to translate acid biology into clinical impact. These include: (1) spatial and temporal quantification of joint pH; (2) multi-omics linkage between pH and cell states; (3) mapping sensor redundancy and signaling cross-talk; (4) clarification of acid source dynamics and buffering; (5) development of pH-informed biomarkers and endotypes; (6) optimization of delivery and safety for acid-targeted therapies; and (7) standardized experimental and clinical trial protocols for OA studies.

Bulk synovial fluid (SF) pH in OA frequently reads near blood, whereas RA shows marked reductions; yet these SF measures can miss tissue-level acidity documented in cartilage and meniscus, underscoring a spatial sampling gap [9,10,11]. Noninvasive acidoCEST-UTE MRI reveals lower pH within OA joint tissues and heterogeneity across regions, but prospective, longitudinal imaging that links pH to progression and flare dynamics is still scarce [9,14]. Standardized protocols that integrate SF, tissue-level imaging, and clinical readouts are needed to define acidotic niches and their temporal evolution in OA [9,10] (Figure 6).

### 7.2. Connecting Acidosis to Cell Programs with Multi-Omics

OA chondrocytes exhibit glycolytic reprogramming under hypoxia and cytokines, and acid-exposed cells engage stress/apoptosis pathways, but integrated pH-controlled transcriptomic, proteomic, and metabolomic maps across stages are limited [16,21,23,26,29,76]. Synovial fluid metabolomics shows glycolysis/lactate signatures, yet datasets rarely include matched tissue pH and cell-state annotation, hampering causal inference [31,32]. Multi-omic atlases under controlled pH are warranted to link acid load to catabolism, inflammation, and senescence in human OA tissues [16,23,29].

### 7.3. Sensor Redundancy and Cross-Talk (GPCRs vs. Ion Channels)

Proton-sensing GPCRs (GPR4, GPR65, GPR68, GPR132) and ion channels (ASIC1a/3, TRPV1) co-exist in joint cells, but their division of labor—by cell type, pH window, and mechanical context—remains undefined in OA [19,20]. GPR68 integrates low pH with membrane stretch, suggesting mechanochemical gating in loaded cartilage, yet in-tissue mapping of G-protein coupling (Gq/11 vs. G12/13) is lacking [20,68]. ASIC1a drives Ca^2+^-dependent stress in chondrocytes, while ASIC3/TRPV1 govern nociception; how these channels interact with pH-GPCR pathways (e.g., NF-κB/MAPK/RhoA) in synovium and cartilage has not been systematically resolved [16,17,18,76].

### 7.4. Acid Sources and Flux: Transporters, Exchangers, and Buffering

Direct OA evidence for lactate/proton transport remains sparse: RA studies implicate MCT4 in SF acidification, but stage-resolved MCT1/4 expression and function in OA synovium/cartilage need definition [34]. Basal pH regulation via Na^+^/H^+^ exchangers and carbonic anhydrases is established in chondrocytes, yet disease-stage changes and their impact on extracellular pH have not been quantified in vivo [36,37,39]. The contribution of subchondral osteoclast acid secretion to osteochondral-junction pH, relative to protease-dominant cartilage erosion, requires compartment-specific measurements [33,42] (Figure 6).

### 7.5. Biomarkers and Endotypes for pH-Directed Therapy

Because bulk SF pH can appear near neutral while tissues are acidic, enrichment strategies are needed to identify “acidotic OA” endotypes most likely to benefit from pH-targeted interventions [9,10]. Imaging biomarkers (acidoCEST-UTE) could guide trial inclusion and serve as pharmacodynamic readouts; however, validation against histology and clinical outcomes is needed [9,14]. Circulating or SF surrogates of tissue acidity—e.g., lactate/pyruvate ratios integrated with transporter expression—have not been standardized across cohorts [31,32] (Figure 6).

### 7.6. Therapeutic Targeting: Selectivity, Delivery, and Safety

Cathepsin-K inhibition shows structural benefit without pain relief, illustrating the need to pair pH-axis drugs with symptom targets or early-stage cohorts [60,89]. For pH-GPCRs, cell-type-specific effects (endothelium vs. cartilage vs. synovium) argue for local delivery and biased/allosteric approaches [62,63,67,78]. ASIC/TRPV1 strategies require peripherally restricted tools to avoid CNS/thermoregulatory liabilities; early intra-articular TRPV1-agonist programs show promise but need longer trials with structural endpoints [91,92,94]. pH-responsive carriers can concentrate therapy in acidic niches, yet translation will depend on manufacturability, payload compatibility, and regulatory acceptance [97,98,99].

### 7.7. Experimental Standards and Clinical Trial Design

Field-wide standards are needed for reporting pH conditions in vitro (media buffering, CO_2_, pHe/pHi readouts), mechanobiology context (strain, compression), and OA stage to ensure reproducibility and comparability across studies [23,29]. Early-phase clinical trials should incorporate pH-aware eligibility (imaging/biomarker enrichment), target-engagement endpoints (e.g., receptor occupancy or ASIC pharmacodynamics), and joint-localized delivery where feasible [14,60,78] (Figure 6).

## 8. Conclusions

OA unfolds within a chemically and mechanically stressed joint, and mounting evidence shows that localized acidosis is a consistent, biologically meaningful feature of this microenvironment. Even when bulk synovial fluid appears near neutral, cartilage, meniscus, and the osteochondral interface harbor acidic niches that reshape cell behavior and protease activity. This acidotic stress arises from convergent sources—hypoxia-driven glycolysis, synovitis and immune metabolism, limits in proton/lactate handling, matrix charge effects, and subchondral bone remodeling—and helps explain why inflammation, matrix loss, and pain so often travel together in OA.

Mechanistically, proton sensors translate pH shifts into pathology. In cartilage, acidosis tilts proteolysis toward cathepsins and triggers ASIC1a-dependent calcium stress, autophagy, senescence, and apoptosis; in afferents, ASIC3 and TRPV1 couple acidity to nociception. In parallel, proton-sensing GPCRs (GPR4, GPR65, GPR68, GPR132) convert extracellular acidity into G_s_/G_q_/G_12–13_ signals that converge on MAPK, NF-κB, CREB, and RhoA/ROCK, influencing chondrocyte catabolism, synovial cytokines, endothelial adhesion, and leukocyte trafficking. These pathways are not peripheral epiphenomena; they sit on the main routes through which OA accumulates structural damage and symptoms.

Therapeutically, the acid axis is tractable. Clinical experience with cathepsin-K inhibition shows that intervening on acid-enabled proteolysis can slow structural progression, even if symptoms require complementary strategies. Preclinical data support modulation of pH-sensing receptors (e.g., GPR68, GPR4), ASIC/TRPV1 targeting for pain and early structure protection, metabolic control (e.g., LDHA) to reduce acid generation, and pH-responsive delivery to confine drug action to the very niches where it is needed. The next leap will come from pH-aware trial design—pairing noninvasive pH imaging or molecular surrogates with cell-type-selective agents and joint-localized delivery and enriching for patients with an “acidotic OA” endotype.

In our view, pH is not a bystander but a modifiable dimension of joint biology. By aligning measurement (spatially resolved pH maps and matched omics) with mechanism (defined proton sensors and transporters) and with therapy (precision delivery and rational combinations), the field can move beyond symptomatic relief toward genuine disease modification in OA.

## Figures and Tables

**Table 1:** Joint pH in OA and RA (compartment-specific).

Study (Year)	Condition	Compartment	Method (Brief)	Mean pH	Range	Notes	Refs.
Jebens and Monk-Jones (1959)	Normal	Synovial fluid (knee)	Aspirated fluid; pH electrode (ex vivo)	7.768 ± 0.044	≈6.5–8.9 (histogram)	Venous blood in same cohort ~7.38; SF > blood on average	[8]
Jebens and Monk-Jones (1959)	OA (knee)	Synovial fluid	Aspirated fluid; pH electrode (ex vivo)	7.549 ± 0.040	Histogram reported	Modest acid shift vs. normal SF	[8]
Roman et al. (2017)	Severe OA (K/L IV; hip/knee)	Synovial fluid	Aspirated at arthroplasty; bench pH meter	7.35	6.80–7.68	Values clustered 7.24–7.50 in most samples	[13]
High et al. (2019)	Mixed knee cohort	Cartilage	acidoCEST-UTE MRI (in vivo)	≈6.60 ± 0.17 *p* = 0.043	NA	Cartilage more acidic than meniscus	[14]
High et al. (2019)	Mixed knee cohort	Meniscus	acidoCEST-UTE MRI (in vivo)	≈6.72 ± 0.16	NA	Meniscus slightly higher pH than cartilage	[14]
High et al. (2019)	Mixed knee cohort	Intra-articular fluid (post-contrast)	acidoCEST-UTE MRI vs. electrode	≈7.22 (iopamidol); ≈7.65 (iohexol)	NA	Agent-dependent fluid estimates	[14]
Lombardi et al. (2022)	Knee OA vs. no-OA	Combined ROIs (cartilage, meniscus, fluid)	acidoCEST-UTE MRI (in vivo)	OA 6.40 ± 0.08; no-OA 7.01 ± 0.26	NA	Each ROI lower in OA; OA meniscus > OA cartilage	[9]
Konttinen et al.	Hip OA (intraoperative)	Articular cartilage (graded)	Microelectrode (surgical)	Normal 7.1 ± 0.4; G1 6.2 ± 0.9; G2 5.7 ± 1.0; G3 5.5 ± 1.0	NA	Acidity increases with damage. G1, G2, and G3 are cartilage damage grades with increasing structural damage	[15]
Goldie and Nachemson (1969)	RA (knee)	Intra-articular cavity (in vivo)	Antimony microelectrode	≈6.6 (down to ≈6.0)	≈6.0–7.3 across individuals	Demonstrates strong in vivo acidosis in RA	[11]

**Table 2:** Therapeutic strategies targeting acidosis/sensing in OA.

Therapy Class	Molecular Target(s)	Rationale (OA/Acidosis Link)	Stage of Evidence	OA Outcome Signal	Key Limitations/Risks	Example Agent(s)	Refs.
Protease inhibition	Cathepsin K (CTSK)	Acid-enabled collagenolysis; cathepsin K degrades type II collagen/aggrecan.	Human phase 2a + preclinical	^†^ Slowed structural progression (qMRI) without clear pain benefit (26 wks).	Structure–symptom dissociation; bone remodeling effects; safety.	MIV-711	[43,60,89]
Proton-sensing GPCR modulation	GPR4 (Gs–cAMP/EPAC)	Acidosis→GPR4 induces endothelial adhesion and inflammatory genes; antagonism suppresses.	Preclinical	GPR4 modulation alters cartilage/synovitis/osteophytes/pain in models.	Vascular/immune effects; specificity; no OA clinical data.	GPR4 antagonists (tool compounds)	[62,63,69]
Proton-sensing GPCR modulation	GPR4 (Gs–cAMP/EPAC)	Acidosis→GPR4, which regulates CXCL12/CXCR7 signaling in chondrocytes	cell/explant/animal	GPR4 antagonism inhibits NF-κB, suppresses collagen degradation.	In vitroSW1353 chondrocyte cell line	NE 52-QQ57	[70]
Proton-sensing GPCR modulation	GPR68/OGR1 (Gq/11, G12/13)	Ogerin (PAM) suppresses IL-1β-induced MMP13 in human OA chondrocytes.	Preclinical (cells; medicinal chemistry)	Hypothesized chondroprotection via reduced MMP13/catabolism.	Cell-type differences; mechanosensitivity; delivery.	Ogerin and analogs	[20,67,78]
Proton-sensing GPCR modulation	GPR65/TDAG8 (Gs–cAMP)	Anti-inflammatory signaling in immune cells; may temper synovitis in acidic niches.	Preclinical	^†^ OA-specific efficacy unknown.	Limited OA pharmacology; immunomodulation risks.	Tool compounds	[64,66]
Proton-sensing GPCR modulation	GPR132/G2A	Proton/lysophospholipid-responsive; sensitizes TRPV1 (pain link).	Preclinical	^†^ Pain-axis modulation plausible; limited OA data.	Ligand ambiguity; cross-talk.	Tool ligands	[20,65,72]
ASIC channel inhibition	ASIC3 (nociceptors)	Proton-driven joint pain via ASIC3; blockade reduces pain and early damage.	Preclinical (rodent OA)	Analgesia and structure-sparing when dosed early.	Peptide delivery; selectivity; human data needed.	APETx2	[17]
ASIC channel inhibition	ASIC1a (chondrocytes)	Acid-induced Ca^2+^ overload and apoptosis; blockade is chondroprotective in vitro.	Preclinical (cells/tissue)	Protects chondrocytes from acid injury.	CNS expression; peptide tools; selectivity.	PcTx-1; early small molecules	[16,50,76]
Proton-sensitive K2P channels	TASK-1/3/2; TREK-1/2; TRAAK	pH- and mechano-modulated K^+^ conductances → membrane potential/Ca^2+^; cartilage mechanobiology	Mechanistic (cartilage cells), emergent OA target class	Candidate chondroprotection via electrophysiology tuning. ^†^	Early stage; pharmacology maturity; specificity	TREK/TASK tool ligands ^†^	[80,81,82,85,86,87]
TRPV1 targeting	TRPV1 (nociceptors; synovium)	Acidic niches sensitize TRPV1; analgesia possible but systemic antagonists cause hyperthermia.	Clinical (antagonists safety) + early OA trials (RTX)	IA RTX shows analgesic signals; systemic antagonists limited by hyperthermia.	Thermoregulation; local ablation risks.	AZD1386; Resiniferatoxin (RTX)	[91,92,93,94]
Metabolic control	LDHA (glycolysis leading to lactate)	LDHA drives lactate/ROS; inhibition reduces OA pathology in vivo.	Preclinical (rodent OA)	Cartilage protection; reduced inflammation.	Systemic effects; delivery.	FX11 (tool)	[35]
Lactate/proton export blockade	MCT1/4 (SLC16A1/A3)	MCTs co-transport lactate + H^+^; inhibition could reduce acid load.	Clinical (oncology) + preclinical (mechanism)	Conceptual; no OA trials yet.	Systemic toxicity; compensations; delivery.	AZD3965	[95,96]
pH-responsive delivery	Stimulus-responsive carriers (intra-articular)	Low pH triggers release; prolongs joint residence; targets acidic niches.	Preclinical (multiple OA models)	Improved cartilage protection/synovial control.	Manufacturing; regulatory path; payload constraints.	pH-responsive HA-MOF NPs; hydrogels	[98,99]

^†^ Non-OA context; mechanistic relevance.

## Data Availability

No new data were created or analyzed in this study. Data sharing is not applicable to this article.
